# Progranulin Knockout Accelerates Intervertebral Disc Degeneration in Aging Mice

**DOI:** 10.1038/srep09102

**Published:** 2015-03-16

**Authors:** Yun-peng Zhao, Qing-yun Tian, Ben Liu, Jason Cuellar, Brendon Richbourgh, Tang-hong Jia, Chuan-ju Liu

**Affiliations:** 1 Department of Orthopaedic Surgery, New York University Medical Center, New York, NY. 10003; 2 Department of Spinal Surgery, Qilu Hospital, Shandong University, Jinan, Shandong 250012, PR China; 3 Department of Orthopaedic Surgery, Jinan Central Hospital, Shandong University, Jinan, Shandong 250012, PR China; 4 Department of Cell Biology, New York University School of Medicine, New York, NY 10016

## Abstract

Intervertebral disc (IVD) degeneration is a common degenerative disease, yet much is unknown about the mechanisms during its pathogenesis. Herein we investigated whether progranulin (PGRN), a chondroprotective growth factor, is associated with IVD degeneration. PGRN was detectable in both human and murine IVD. The levels of PGRN were upregulated in murine IVD tissue during aging process. Loss of PGRN resulted in an early onset of degenerative changes in the IVD tissue and altered expressions of the degeneration-associated molecules in the mouse IVD tissue. Moreover, PGRN knockout mice exhibited accelerated IVD matrix degeneration, abnormal bone formation and exaggerated bone resorption in vertebra with aging. The acceleration of IVD degeneration observed in PGRN null mice was probably due to the enhanced activation of NF-κB signaling and β-catenin signaling. Taken together, PGRN may play a critical role in homeostasis of IVD, and may serve as a potential molecular target for prevention and treatment of disc degenerative diseases.

Degenerative disc disease (DDD) is one of the most prevalent degenerative diseases in aging population in which intervertebral disc (IVD) undergoes extensive morphological as well as biomechanical changes, and usually manifests clinically in patients with lower back pain^1^,^2^. The mechanisms involved in this degenerative process have not been fully understood, and therapies are mainly palliative. A majority of the researches concerning this issue focus on: the relationship between bone quality, bone metabolism and IVD degeneration, bony tissue formation in IVD and abnormal change of trabecular bone quality in adjacent vertebra^3^. Moreover, cartilage degeneration is extensively investigated, because cartilage is a major structural component of normal IVD, and the loss of proteoglycan, a dominant component of cartilage, is a feature of disc degeneration^4^,^5^,^6^.

Progranulin (PGRN) is a pleiotropic growth factor with a plethora of functions. PGRN is expressed in various cells and plays a critical role in many physiological and disease processes such as: wound healing^7^, tumorigenesis^8^ and inflammation^9^,^10^,^11^. Studies have also found that low levels of PGRN can cause degenerative diseases of the nervous system in both human and mice^9^,^12^,^13^. We previously reported that PGRN was expressed in human articular cartilage, and its level was significantly elevated in cartilage of patients with osteoarthritis and rheumatoid arthritis^14^. In addition, PGRN also plays a crucial role in chondrocyte proliferation^15^, differentiation and endochondral ossification of growth plate during development^16^. PGRN antagonized tumor necrosis factor-α (TNF-α) through its ability to bind to TNF receptors. It was also documented that PGRN exhibits anti-inflammatory function within inflammatory arthritis models^17^. Recently, we found that PGRN play a critical role in maintaining homeostasis of cartilage and protect against osteoarthritis^18^,^19^. Herein we examined the expression pattern of PGRN in IVD tissue of human and mice under physiological and degenerative conditions, and determined the potential effects of PGRN deficiency on IVD degeneration as well as the alteration of signaling pathways during aging process.

## Results

### PGRN is expressed in both human and murine IVD tissue and its levels are elevated in murine IVD during aging

**Figure 1:**
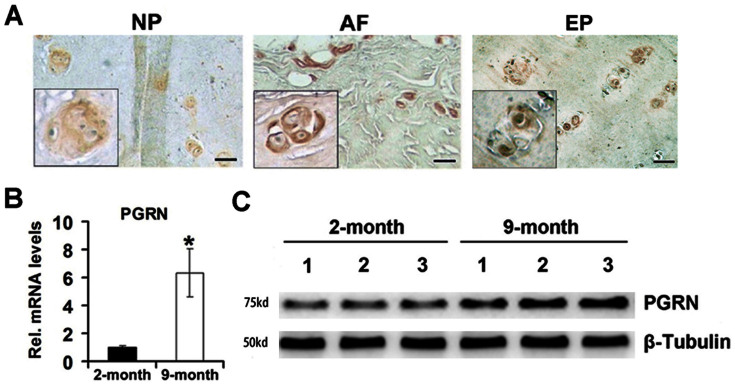
PGRN is expressed in disc tissues of both human and mice and its level is elevated in the mouse IVD through aging. (A) PGRN was detectable in the extracellular matrix of the cell clusters formed in NP (left panel), AF (middle panel) and EP (right panel) from degenerated discs. Samples from disc degeneration patients (n = 7) were collected and were stained with anti-PGRN antibody (brown), then counterstained with methyl green (green). Representative pictures are shown. The inserts indicate higher magnification views of cell clusters. Scale bar, 25 μm. (B) RNA level of PGRN in 2-month and 9-month old mice (n = 3, respectively), assayed by real-time PCR. The relative unit of PGRN expression for 2-month old mice was set to 1. *p < 0.05. (C) Protein level of PGRN in IVD of 2-month and 9-month old mice, assayed by Western Blotting. Total IVD extracts from 2-month and 9-month old mice (n = 3, respectively) were resolved using 10% SDS-PAGE and probed with anti-PGRN and anti-β-tubulin (internal control) antibodies.

To investigate the potential involvement of PGRN in disc degeneration of human being, we examined its expression pattern in IVD tissue disc degeneration patients. Immunohistochemistry results demonstrated PGRN was detectable in cell clusters formed in nucleus pulposus (NP) (Figure 1A, left panel), annulus fibrosus (AF) (Figure 1A, middle panel) and end plate (EP) (Figure 1A, right panel) structures of IVD. High-resolution analysis (Figure 1A, inserts) detected that in the cell clusters formed in all mentioned three parts of IVD tissue, PGRN was specifically expressed in the extracellular matrix and cytoplasm of the cell clusters, which implied a role of PGRN during the process of IVD degeneration. To investigate the expression pattern of PGRN in the mouse IVD during aging process, total IVD tissue was collected from 2- and 9-month old WT mice, and real time PCR as well as western blotting were performed (n = 3 for each group). As shown in Figure 1B and 1C, both mRNA and protein levels of PGRN were elevated in 9-month-old IVD compared with 2-month old group.

### PGRN knockout mice develop ectopic bone formation and an early onset of degeneration in IVD cartilage

**Figure 2:**
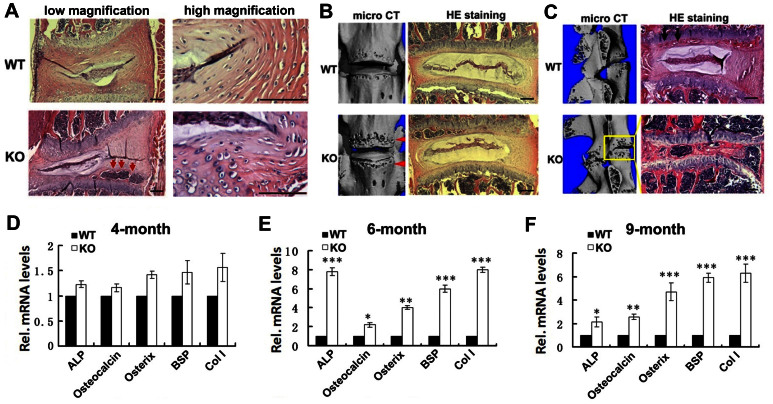
Knockout of PGRN leads to abnormal bony tissue formation and degeneration in IVD during aging. (A) New bone formation (low magnification, red arrows) and change of cell type and density (high magnification) in IVD tisssue of PGRN−/− mice. IVD from 4-month old WT and PGRN−/− mice were examined through hematoxylin and eosin (HE) staining. Representative images for both low (left panel) and high (right panel) magnifications are shown. (B) New bone formation in IVD of 6-month old PGRN−/− mice (red arrows). IVD samples from 6-month old WT and PGRN−/− mice were collected then micro CT and HE staining were performed. (C) In 9-month old groups, WT mice showed new bone formation in EP (black arrows), while severe new bone formation and narrowing of intervertebral space (yellow box) were observed in PGRN−/− mice, assayed by micro CT and HE staining. (D, E, F) Elevated ALP, osteocalcin, Osterix, BSP, Col1 levels in IVD of 6- and 9-month old PGRN−/− mice, assayed by real-time PCR (n = 3, respectively). RNA was extracted from IVD of all indicated groups, and real-time PCR was performed. The values are mean ± SD of 3 independent experiments. * p < 0.05, ** p < 0.01 and *** p < 0.005 vs. WT group. Scale bar, 100 μm.

To determine the role of endogenous PGRN in maintaining integrity of IVD, we assessed the morphology of the IVD tissues from 4-, 6- and 9-month-old WT and PGRN−/− mice. At 4 months of age, early onset of degeneration was observed in the IVD tissue of PGRN−/− group. The morphology of the cartilage at this stage showed disorganization as well as newly formed bone was present in PGRN−/− mice (Figure 2A, left panels). The normal cell phenotype was replaced by degenerative chondrocyte-like cells (Figure 2A, right panels). In 6-month-old mice, new bone formation in IVD tissue was detected via micro CT and histology (Figure 2B), and 9-month-old PGRN−/− mice showed narrowing of intervertebral space together with very severe bony tissue formation in IVD (Figure 2C). Levels of osteoblastic marker genes, including alkaline phosphatase (ALP), osteocalcin, osterix, collagen I (Col I) and bone sialoprotein (BSP) were analyzed through real-time PCR (n = 3 for each group), and the result revealed that expressions of these markers were significantly higher in PGRN−/− mice in both 6- and 9-month old group (Figures 2D, 2E and 2F), which were consistent with acceleration of new bone formation during the aging process observed within these mutant mice by micro CT assay and HE staining.

**Figure 3:**
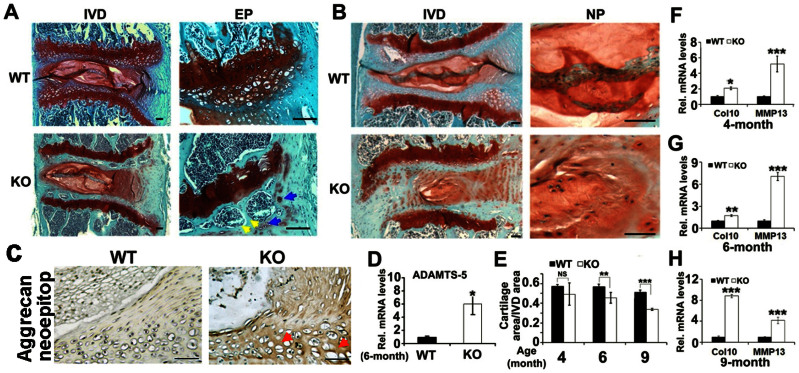
PGRN deficiency leads to cartilage defects during aging. (A) 6-month old PGRN−/− mice revealed formation of cell clusters (blue arrows) and new bone (yellow arrows) in IVD, assayed by Safranin O staining. (B) Severe degeneration in IVD of 9-month old PGRN−/− mice, in which the boundary between NP and AF became unclear (left panel), normal NP structure was replaced by degenerative fibrocartilage structure and clefts were formed (right panel). (C) Enhanced degradation of aggrecan in 6-month old PGRN−/− mice, detected by immunohistochemistry for new-epitope of aggrecan. PGRN−/− mice revealed more degradation of aggrecan compared with WT littermates, indicated by brown color distributed in extracellular region (red arrows). (D) Enhanced ADAMTS-5 level in IVD of PGRN−/− mice, assayed by real time PCR (n = 3, respectively). RNA from 6-month old WT and PGRN−/− IVD was extracted and analyzed with real-time PCR. (E) Exaggerated loss of cartilage structure in IVD of PGRN−/− mice, assayed by histomorphometric analysis. (F, G, H) Elevated MMP13 and Col10 mRNA levels in IVD of PGRN−/− mice, demonstrated by real-time PCR (n = 3, respectively). The values are the mean ± SD of 3 independent experiments. * p < 0.05, ** p < 0.01 and *** p < 0.005 vs. WT group. Scale bar, 100 μm.

To assess the loss of proteoglycan and disorder of cartilage structure in intervertebral disc, we performed Safranin O staining. In 6-month old PGRN−/− mice, loss of proteoglycan was severe in the endplate cartilage, accompanied by newly formed bone, and high-resolution analysis showed that cell clusters were formed in EP (Figure 3A). In 9-month old WT mice, loss of proteoglycan and newly formed bone were detectable in EP tissue. In 9-month old PGRN−/− mice, disorder of AF was severe with extensive loss of proteoglycan, alteration of cell type and cleft formation in addition to degeneration changes in the EP and the boundary between NP and inner AF became less clear (Figure 3B, left panel). Moreover, degenerative fibrocartilage, chondrocyte-like cells, mucous degeneration and clefts were present in NP tissue of PGRN−/− mice, which were absent in WT littermates (Figure 3B, right panel). To verify the degradation of aggrecan, immunohistochemistry for neo-epitope of aggrecan was performed in 6-month old WT and PGRN−/− mice, and dramatically stronger signal was observed in IVD of PGRN−/− mice (Figure 3C). To investigate the accelerated aggrecan degradation in IVD of PGRN−/− mice, we collected RNA from IVD of WT and PGRN−/− mice, and performed real time RT-PCR to assess level of ADAMTS-5. Figure 3D indicates that ADAMTS-5 level was dramatically elevated in PGRN−/− group compared to the WT controls, which may explain the enhanced degradation of aggrecan in PGRN−/− group. As the feature of cartilaginous structure is the proteoglycan matrix and cartilage cell, based on the Safranin O staining of intervertebral disc, percentage of cartilaginous area in IVD was assessed with histomorphometric software, and data demonstrated that although there was no statistical significance in 4-month group, in 6- and 9-month old groups PGRN−/− mice exhibited significantly lower cartilage area percentage compared with WT littermates (Figure 3E). To further confirm the degeneration of cartilage tissue in IVD, we performed real time RT-PCR (n = 3 for each group) to assess levels of Col10 and MMP13. Expressions of both Col10 and MMP13 were significantly higher in all three aged groups of PGRN−/− mice compared to the corresponding WT controls (Figures 3F, 3G and 3H), clearly indicating that absence of PGRN leads to accelerated cartilage degeneration.

### PGRN knockout mice display significantly higher pathological scores in the IVD tissue

**Figure 4:**
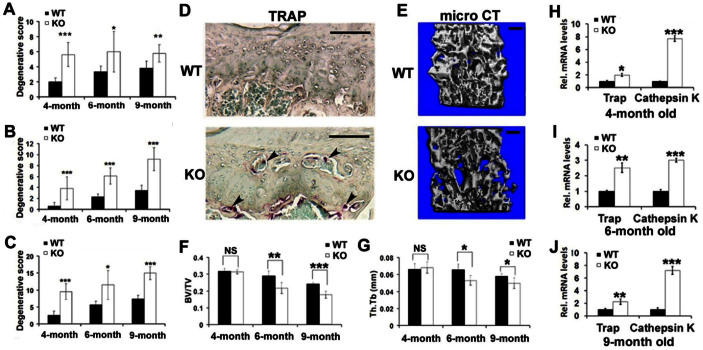
Deficiency of PGRN leads to higher histological grade for IVD, and enhances osteoclast activity in IVD and adjacent vertebra. (A) Degenerative scores of EP in PGRN−/− mice were significantly higher than those of WT mice. (B) PGRN deficiency led to significantly higher degenerative score of AF/NP compared with WT littermate in all three aged groups. (C) Degenerative score of total IVD also showed statistical difference between WT and PGRN−/− mice in each age group. * p < 0.05, ** p < 0.01 and *** p < 0.005 vs. WT group. (D) Higher activity of osteoclast in IVD and adjacent vertebra of 6-month old PGRN−/− mice (black arrows), determined by TRAP staining. (E) Osteoporosis change in trabecular bone of L4 vertebra in 6-month old PGRN−/− mice, assayed by micro CT. (F and G) BV/TV and Tb.Th were significantly lower in trabecular bone of L4 vertebra in 6-month and 9-month old PGRN−/− mice, assayed by micro CT (n = 5 for each group). (H, I, J) Elevated gene expressions of TRAP and Cathepsin K in IVD of PGRN−/− mice, assayed by real-time PCR (n = 3, respectively). The values are the mean ± SD of 3 independent experiments. * p < 0.05, ** p < 0.01 and *** p < 0.005 vs. WT group. Scale bar, 50 μm.

On the basis of histology, the histological grading system was used as proposed by Boos et al^20^. According to the parameters mentioned, the scoring and statistical analysis revealed that in the EP tissue, PGRN−/− mice showed a significantly higher score as early as 4-month old in comparison of WT mice (Figure 4A). However, EP score of PGRN−/− mice underwent no significant change with aging process (4 M vs. 6 M, p = 0.8445; 4 M vs. 9 M, p = 0.8019; 6 M vs. 9 M, p = 0.7552). This may suggest that the EP sustained quite severe degeneration during the early stage in the mutant mice and remained at that increased level during aging. In the AF/NP tissue, PGRN−/− mice gave rise to higher scores within each age group (Figure 4B). In 4- and 6-month old mice, we did not observe severe degeneration in NP of both genotypes; while in 9-month old group, PGRN−/− mice exhibited degeneration of fibrocartilage, change of cell type to cartilage-like cells, mucous degeneration and cleft formation in NP, which further contributed to the difference of histological score between WT and PGRN−/− mice (Figure 4B). Then we put the scores from AF/NP and EP together and found consistent trend with scores respectively (Figure 4C), which suggested that absence of PGRN led to significant degeneration in IVD tissue. There was no significant difference of score between two observers.

### PGRN knockout mice display augmented osteoclastogenesis for erosion of EP and trabecular bone of vertebra

To further study the role of PGRN in integrity of spine, we tested osteoclastogenesis in WT and PGRN−/− mice. TRAP staining of 6-month old spine showed remarkably elevated osteoclast activity within both the newly formed bone tissue (in EP cartilage) and the trabecular bone (beneath growth plate of lumbar vertebra) in PGRN−/− mice, which was not present in the WT littermates (Figure 4D). To verify the osteoclast activity in spine, we used micro CT to analyze the trabecular bone in L4 vertebra of 6-month old WT and PGRN−/− mice, and found that PGRN deficient mice exhibited lower quality of trabecular bone (Figure 4E). The parameters of trabecular bone through micro CT, bone volume/tissue volume/ (BV/TV) and thickness of trabecular bone (Th.Tb) were significantly lower in 6- and 9-month old PGRN−/− mice, which implied accelerated osteoporosis in the vertebra of these mice (Figures 4F and 4G). According to micro CT data, there was no significant difference in 4-month old group between genotypes. Then we examined the expressions of the marker genes concerning osteoclastogenesis, including TRAP and Cathepsin K through real time RT-PCR (n = 3 for each group), and found that higher level of these genes were observed in each PGRN−/− aged group (Figures 4H, 4I and 4J).

### PGRN knockout mice exhibit enhanced activation of NF-κB signaling in IVD

**Figure 5:**
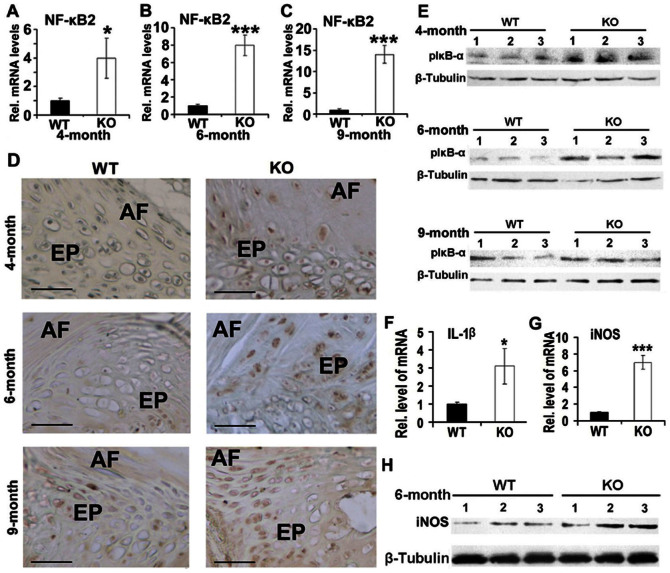
PGRN deficiency leads to augmented NF-κB signaling pathway in IVD. (A, B, C) Elevated NF-κB2 expression in IVD of PGRN−/− mice, assayed by real-time PCR. RNA was extracted from IVD of all indicated groups, real-time PCR was performed. (D) Enhanced Phosphorylated IκB-α (pIκB-α) signaling in EP cells (black arrows) of PGRN−/− mice, tested by immunohistochemistry. IVD sections from 4-, 6- and 9-month old WT and PGRN−/− mice were stained with anti-pIκB-α antibody (brown) and counterstained with methyl green (green). Representative pictures are shown. Scale bar, 50 μm. (E) Increased expression of pIκB-α in IVD of PGRN−/− mice, assayed by Western Blotting. Total protein extracts were collected from 3 mice of each aging group and Western Blotting was performed. (F, G) Elevated IL-1β, iNOS levels in IVD of PGRN−/− mice, assayed by real-time RT-PCR (n = 3, respectively). RNA from 6-month old WT and PGRN−/− IVD was extracted, followed by real-time RT-PCR. (H) Increased iNOS expression in IVD of PGRN−/− mice, assayed by Western Blotting. Total IVD protein extracts were collected from three 6-month old WT and PGRN−/− mice, and Western Blotting was performed. The values are the mean ± SD of 3 independent experiments. *p < 0.05, ** p < 0.01 and *** p < 0.005 vs. WT group.

Our recent finding that PGRN inhibited TNF mediated activation of NF-κB signaling pathway^21^, together with the reports that NF-κB signaling played a critical role in IVD degeneration^22^, promoted us to determine whether PGRN deficiency affected NF-κB signaling that in turn contributed the IVD degeneration. To investigate the alteration of NF-κB signaling expression in the absence of PGRN, NF-κB2 level was measured using real time RT-PCR (n = 3 for each group). As revealed by Figures 5A, 5B and 5C, NF-κB2 level was significantly higher in IVD of all three PGRN−/− aging groups. To further determine the effects of PGRN deficiency on the activation of NF-κB signaling, immunohistochemistry was performed for phosphorylation of IκB-α, an inhibitor of NF-κB activity in IVD, and 4-, 6- and 9-month old PGRN−/− mice demonstrated remarkably higher signal of pIκB-α around nuclei of cells in EP compared with WT controls (Figure 5D); in addition, total IVD extracts were collected from both WT and PGRN−/− mice and western blotting was performed. As shown in Figure 5E, the level of pIκB-α was elevated in all PGRN−/− aging groups. The combination of this experimental data show that a loss of PGRN results in augmented NF-κB signaling in IVD. Nitrous Oxide (iNOS) and interlukin-1β (IL-1β) are target genes of NF-κB signaling which have been reported to be involved in IVD degeneration^23^. To determine the altered expression level of iNOS in deficiency of PGRN, RNA extracts were collected from IVD of 6-month old WT and PGRN−/− mice. The RNA level of IL-1β and iNOS were measured through real time PCR (n = 3 for each group), and IVD of PGRN−/− mice showed increased level of both target genes (Fig. 5F and 5G). In addition, protein level of iNOS was evaluated through western blot analysis, and IVD of PGRN−/− mice exhibited increased iNOS expression in protein level (Fig. 5H). Collectively, these data suggest that both expression and activity of NF-κB signaling was enhanced in IVD of PGRN−/− mice.

### PGRN knockout mice display increased expression of β-catenin and its downstream target genes in IVD

**Figure 6:**
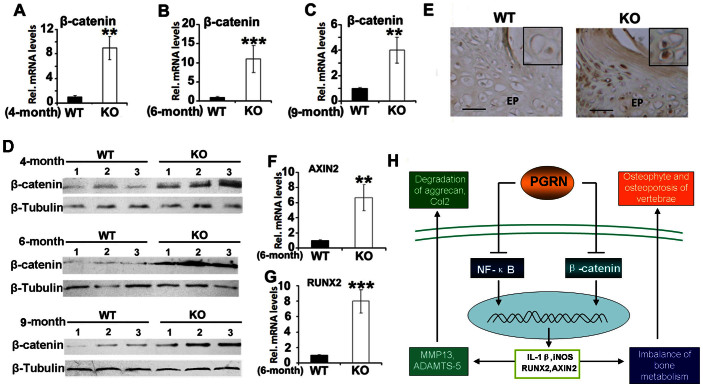
Expressions of β-catenin and its target genes are enhanced in IVD of PGRN−/− mice. (A, B, C) Enhanced mRNA level of β-catenin in IVD of PGRN−/− mice, assayed by real-time PCR (n = 3, respectively). RNA was extracted from IVD of 4, 6, 9-month old WT and PGRN−/− mice, followed by real-time PCR. (D) Increased expression of β-catenin in IVD of PGRN−/− mice. Total IVD protein extracts were collected from 3 mice of each group, and assayed by Western Blotting with anti-β-catenin, anti-β-tubulin (internal control) antibodies. (E) PGRN−/− mice revealed stronger β-catenin signal and the nuclear translocation (inserts) in IVD cells, determined by immunohistochemistry. IVD sections from 6-month old WT and PGRN−/− mice were stained with anti-β-catenin antibody (brown) and counterstained with methyl green (green). Representative pictures are shown. Scale bar, 50 μm. (F, G) Elevated mRNA levels of AXIN2, RUNX2 in IVD of PGRN−/− mice, measured by real-time PCR (n = 3, respectively). RNA was collected from IVD of 6-month WT and PGRN−/− mice, followed by real-time PCR. (H) A proposed model for explaining the protective role of PGRN in the course of IVD degeneration. The values are the mean ± SD of 3 independent experiments. *p < 0.05, ** p < 0.01 and *** p < 0.005 vs. WT group.

The fact that Wnt/β-catenin signaling pathway is an another pathway known to play an important role in IVD degeneration action^24^. together with the recent report that loss of PGRN resulted in increased expressions of Wnt signaling molecules in neural system^25^,^26^, led us to examine whether Wnt/β-catenin signaling pathway is involved in the PGRN-deficiency mediated IVD degeneration. For this purpose we first examined the effects of PGRN deletion on β-catenin expression in IVD. Briefly, IVD from WT and PGRN−/− mice of indicated ages were harvested and total RNA and protein were extracted for real time RT-PCR and western blotting assay, respectively. As shown in Figures 6A, 6B and 6C, mRNA level of β-catenin was significantly higher in IVD of all PGRN−/− groups (n = 3 for each group). Moreover, the western blot results revealed that β-catenin protein levels are elevated in PGRN−/− mice when compared with WT groups (Figure 6D). Additionally, immunohistochemsitry of β-catenin was performed in IVD of 6-month old WT and PGRN−/− mice. As shown in Figure 6E, β-catenin signal was stronger and more nuclear translocation of β-catenin was observed in IVD tissue of PGRN−/− mice. Collectively, this set of assays suggested that Wnt/β-catenin signaling pathway is probably involved in PGRN-knockout induced osteoblastogenesis and abnormal bone formation observed in PGRN-knockout IVDs (Figure 2). To further investigate the activity of Wnt/β-catenin signaling, expression levels of downstream target genes including Axin2 and RUNX2 in IVD of 6-month old WT and PGRN−/− mice were measured through real time RT-PCR (n = 3 for each group). Figure 6F and 6G determined that Axin2 level and RUNX2 level was significantly higher in PGRN−/− IVD, suggesting the activation of Wnt/β-catenin signaling pathway. On the basis of the present study, Figure 6H presented a proposed model for the role of PGRN in IVD degeneration. PGRN protects against IVD degeneration through at least two pathways. Firstly, PGRN inhibited NF-κB signaling pathway mediated induction of its target genes including ADAMTS (e.g. ADAMTS-5), MMPs (e.g. MMP13) and cytokines (e.g. IL-1β), and protected against inflammatory osteoclastogenesis and destruction of cartilage structure in IVD. Secondly, PGRN impaired Wnt/β-catenin signaling induced downstream molecules such as RUNX2, and fought against new bone formation in cartilaginous tissue of IVD.

## Discussion

PGRN has been known to play a critical role in endochondral ossification during embryo development, and to be expressed in osteoblasts^16^,^27^. In the present study, we found new bone formation in the EP of PGRN−/− mice as early as 4-month old, together with significantly higher levels of osteoblast marker genes, which indicated disorder of bone anabolism in IVD of these mice with aging. We also observed that osteoclast activity was also elevated in each PGRN−/− aged group. This was manifested by more TRAP+ cells in the trabecular bone of the vertebra and ectopic bone formation within the EP, osteoporosis change in trabecular bone of vertebra and elevated levels of osteoclast marker genes including TRAP and Cathepsin K. We reported that PGRN protected bone from resorption in inflammatory arthritis model^21^. Additionally a deficiency of PGRN led to more severe osteoporosis after ovariectomized operation, and administration of recombinant PGRN protein attenuated this process (Tang and Liu, unpublished data). This data shows that PGRN functions in the regulation of osteoclastogenesis, and might explain why accelerated level of osteoporosis occurred within the vertebra of PGRN−/− mice. Moreover, bony tissue formation in IVD and abnormal change of trabecular bone quality in adjacent vertebra are considered involved in IVD degeneration^3^. Collectively, these data suggest that loss of PGRN may lead to defects in bone metabolism of the spine, which accelerate degeneration of IVD.

Proteoglycan is a main constituent of cartilaginous structure including articular cartilage and IVD, and plays an indispensible role in IVD^8^. Proteoglycan loss within the matrix is one of the universal hallmark features of disc degeneration^8^. We observed that proteoglycan loss was dramatically exaggerated in PGRN−/− mice with aging, especially for cartilaginous EP and AF. This suggests enhanced degeneration of cartilage structure in PGRN−/− mice. One possible reason was that PGRN was protective for cartilage matrix and chondrocyte function, as PGRN was reported to promote chondrocyte proliferation, differentiation and cartilage repair in animal models^15^. It has been well established that the degradation of aggrecan, a key matrix protein, is a parameter for IVD degeneration^28^. Here we observed that deficiency of PGRN led to the destruction of cartilage structure and more severe degradation of aggrecan in IVD in vivo. Moreover, ADAMTS-5 level was elevated in IVD of PGRN−/− mice. ADAMTS-5 functions as an aggrecanase in mice, and plays a pivotal role in progression of IVD degeneration^29^. By using an antibody that specifically identifies neoepitope of aggrecan degradation, we found enhanced aggrecan degradation in PGRN−/− mice. These data indicate that PGRN also plays a chondroprotective role in IVD through protecting against matrix degradation. Additionally, PGRN was known to inhibit cartilage degradation mediated by ADAMTS-7 and ADAMTS-12^14^. Recently, it was reported that ADAMTS-7 and ADAMTS-12 are also expressed in rat IVD tissue and their levels were elevated during disc degeneration^5^. In the current study, the expression of MMP13 was significantly higher in each group of PGRN−/− IVD tissue. MMP13 is involved in cartilage degradation and has been used as one of the markers for degeneration of both articular cartilage and IVD^30^. Data from the murine models also revealed that suppression or inhibition of MMP13 can attenuate the degenerative process^31^. Collagen type 10 (Col10) is a marker for cartilage degeneration and its level was also used to monitor the severity of disc degeneration^32^. Collectively, our data demonstrated that absence of PGRN leads to abnormal levels of degeneration-related molecules and severe loss of cartilage matrix through aging.

Extensive studies have found that aging plays a critical role in homeostasis of both articular cartilage and IVD^33^. In the present study, we used longitudinal analysis to evaluate the degeneration of IVD during aging process. The histological grading system for mice disc degeneration mainly focuses on new bone formation and degeneration of cartilage structure. In the EP, the histological score of mutant group was significantly higher from 4-month old, but was not dramatically changed with aging. This may suggest that EP undergoes the degeneration process first and reached a high level of degeneration at relatively young age. On the other hand, the cartilage/IVD area were similar between 4-month old WT and PGRN−/− mice, this may indicate the fibrosis and bone turnover in EP at this age remain at a low level. The expression of bone markers such as ALP, osteocalcin, BSP, osterix and Col 1 were similar between 4-month old WT and PGRN−/− mice, while the expression of chondrocyte hypertrophy and osteoclast marker genes were higher in 4 month old PGRN−/− mice, the result may indicate that the aspects of degeneration did not show up parallel, the hypertrophy and osteoclastogenesis took place ahead of bone turnover. In NP and AF, however, there was remarkable change during aging for both WT and PGRN−/− mice. The result might indicate the time interval between the change of biomarkers and morphological change, which might cause the unparallel signs of degeneration in different experiments.

There is minimal blood supply to the IVD, necessitating diffusion of nutrients through the cartilaginous EP^34^. Therefore, the intact endplate is crucial for normal function of IVD. In this study, we observed that degeneration process occurred in EP tissue as early as 4-month old in PGRN−/− mice. This change may further accelerate the degeneration of AF and NP and leads to complete disc herniation, and spondylosis, because degeneration and the newly formed bone tissue in EP would seriously affect the diffusion process of nutrient source for AF and NP^35^. In the present study, we found that PGRN was not detectable in mice NP tissue, but 9-month old PGRN−/− mice showed more severe degeneration compared with WT littermates. This is probably because that deficiency of PGRN directly disturbs normal function of EP and AF through promoting cartilage degeneration, which subsequently resulted in nutrient deficiency of NP. Taken together, absence of PGRN might affect homeostasis of IVD both directly and indirectly, in the course of IVD degeneration process.

With degenerated IVD from human patients, we found that while PGRN was certainly expressed in the extracellular matrix of cell clusters formed in AF and NP of disc degeneration patients, suggesting a possible role for PGRN in the IVD degeneration. Furthermore, the level of PGRN was elevated during aging in WT mice. This is consistent with our previous report that PGRN level was dramatically elevated in OA and RA patients, and PGRN played a protective role in cartilage destruction^14^,^17^,^18^. It is possible that the elevation of PGRN expression in IVD was secondary to the elevation of inflammatory cytokines which accelerated degeneration process and has a protective role, but the level of PGRN was still not high enough to neutralize or reverse the degenerative factors.

PGRN inhibits TNF-α activity through competitively binding to TNFR1/2 and inactivates NF-κB signaling^21^. This may explain the role of PGRN in the homeostasis of IVD and vertebra during aging. TNF-α is a key inflammatory cytokine and plays a crucial role for inflammatory arthritis, in which it exaggerates cartilage degradation and bone resorption^36^. Anti-TNF inhibitors have been accepted as clinically effective biologics for treating various kinds of inflammatory diseases including rheumatoid arthritis^37^. TNF-α expression has also been reported in human IVD tissue, and studies also demonstrated that TNF-α is involved in IVD degeneration^38^. In addition, anti-TNF-α therapy was reported to be effective to protect disc degeneration^39^,^40^. The NF-κB pathway plays a critical role in mediating TNF-α activity. The NF-κB signaling pathway was known to be a key mediator of age-dependent disc degeneration. Studies have shown that stimulation of NF-κB signaling can accelerate, while inhibition of this signaling can attenuate, disc degenerative diseases associated with aging^41^,^42^. Phosphorylation of IκB-α is a commonly tested parameter for the activation of NF-κB signaling pathway. In our current study, we found that the level of pIκB-α was dramatically elevated in PGRN−/− IVD in each aged group. Moreover, iNOS is a target gene of NF-κB signaling pathway which controls the synthesis of NO, and is involved in IVD degeneration. In this study, iNOS was tested and PGRN−/− IVD presented higher iNOS expression both in RNA level and protein level. Collectively, these data suggested excess activation of the NF-κB signaling pathway in the absence of PGRN may lead to the accelerated IVD degeneration in aging.

The β-catenin signaling pathway has been found to play a critical role in development, metabolism and degeneration of IVD^43^,^44^. It has been found that activation of β-catenin induces IVD degeneration^24^. Recently, it was reported that PGRN interacts with β-catenin signaling pathway in neural system^25^,^26^. Additionally, knockout of PGRN leads to dramatically higher level of expression of β-catenin in primary murine chondrocytes (Zhao and Liu, unpublished data). In the present study, we observed that β-catenin was markedly elevated in both mRNA and protein levels, and the nuclear translocation was also observed in PGRN−/− IVD. It has been reported that activation of β-catenin upregulates its downstream molecule RUNX2, which subsequently exaggerates the IVD degeneration phenotype^45^. In this study, downstream target genes of β-catenin signaling pathway, Axin2 and RUNX2, were expressed in a higher level of PGRN−/− IVD when compared with WT littermates suggesting the activity of β-catenin signaling pathway was also enhanced. Taken together, this implies that a protective role of PGRN in IVD may occur through interacting with the β-catenin signaling pathway. Collectively, PGRN plays an important role in maintaining the integrity of IVD during aging possibly by suppressing NF-κB signaling and β-catenin signaling pathways, which in turn prevents abnormal bone metabolism as well as degeneration of cartilage-like tissue in spine.

## Methods

All the following methods were carried out in accordance with the approved guidelines.

### Mice

All animal studies were performed in accordance with institutional guidelines and approval by the Institutional Animal Care and Use Committee of New York University. The generation and genotyping of PGRN deficient mice have been described previously^17^. 2-, 4-, 6- and 9-month old WT and PGRN−/− mice were used for these experiments.

### Immunohistochemistry

Seventeen IVD samples from patients with disc degeneration were harvested with approval of Institutional Review Boards (IRB#2852 from Sutter Medical Center in California). Besides, IVD tissue from 2-, 4-, 6- and 9-month old WT mice were harvested and fixed in 4% PBS buffered paraformaldehyde at 4°C overnight for immunohistochemistry. After the tissue was dehydrated and embedded in paraffin, 6-μm sections were cut. Thereafter, sections were deparaffinized by xylene immersion, rehydrated by graded ethanol, and treated with 0.1% trypsin for 30 minutes at 37°C. After blocking in 20% goat serum for 60 minutes at room temperature, sections from human IVD were incubated with anti-PGRN polyclonal antibody (1:100 dilution; Santa Cruz Biotechnology), and sections from 6-month old mice were incubated with anti-neoepitope of aggrecan (1:100 dilution;Millipore, Cat. No: AB8135), anti-phosphorylated IκB-α (pIκB-α) (1:100 dilution; Santa Cruz Biotechnology; Cat. No. SC-101713) or anti-β-catenin polyclonal antibody (1:100 dilution; Santa Cruz Biotechnology; Cat. No. SC-1496) at 4°C overnight, followed by incubation with a horseradish peroxidase–conjugated secondary antibody for 60 minutes at room temperature. The signal was detected using the Vector Elite ABC Kit (Vectastain; Vector).

### Histological scoring for degeneration of IVD

The anatomy of IVD was instructed as previously reported^46^, and the degeneration of L3–L4 IVD was scored according to the classification system proposed by Boos et al^20^. This was a classification system for grading the histological features of age-related changes in the lumbar disc. Histological gradings were performed separately on nucleus pulposus (NP)/annulus fibrosus (AF), and endplate (EP). This classification system is based on an extensive semiquantitative histological analysis (NP/AF 0–22, EP 0–18, total 0–40). With this scoring system, a higher score indicates a more severe stage of disc degeneration. In this study we took 26 sections of WT mice (2-month old, n = 6; 4-month old, n = 8; 6-month old, n = 6; 9-month old, n = 6), and 32 sections of PGRN−/− mice (2-month old, n = 6; 4-month old, n = 8; 6-month old, n = 8; 9-month old, n = 10).The sections underwent double blind examinations by 2 authors independently (Y. Z and B. R).

### Micro-CT

Prior to histological processing, paraformaldehyde-fixed spines were evaluated with micro-CT using a Scanco vivaCT40 cone-beam scanner (SCANCO Medical, Switzerland) with 55 kVp source and 145 μAmp current. 6 mice of each genotype and aged group were used in micro-CT assay. We then scanned the spines at a resolution of 10.5 μm. The scanned images from each group were evaluated at the same thresholds to allow 3-dimensional structural reconstruction of each sample. The space between vertebral sections, which implies height of intervertebral disc; the trabecular bone inside the L4 vertebra and the newly formed bone at IVD tissue were analyzed through structural reconstruction.

### Real-time RT-PCR

Total RNA was extracted from L2–L4 IVD tissue using an RNeasy kit (Qiagen, Valencia, CA, USA). Reverse transcription was performed using a RT-for-PCR kit (Qiagen, Valencia, CA) following the manufacturer's protocol. Reactions were performed in a 20-μl SYBR Green PCR volume in a 96-well optical reaction plate formatted in the 7300 Sequence Detection System (Applied Biosystems, Foster City, CA, USA). Sequence-specific primers used within the present study are listed as follows 5′-AACCTATGCCCGTTTCCTCT-3′ and 5′-CCACACATTTCTCCCTCTCC-3′ for AXIN2, 5′-TGATGACACTGCCACCTGTG-3′ and 5′-ACTCTGGCTTTGGGAAGAGC-3′ for RUNX2, 5′-AATCTCACAGCAGCACATCA-3′ and 5′-AAGGTGCTCATGTCCTCATC-3′ for IL-1β, 5′-ACAGGAGGGGTTAAAGCTGC-3′ and 5′-TTGTCTCCAAGGGACCAGG-3′ for iNOS, 5′-TGGTGGAGCAGCAAGAGCAA-3′ and 5′-CAGTGGACAGTAGACGGAGGAAA-3′ for PGRN, 5′-ACACCTTGACTGTGGTTACTGCTGA-3′ and 5′-CCTTGTAGCCAGGCCCGTTA-3′ for ALP, 5′-CTTGAAGACCGCCTACAAAC -3′ and 5′-GCTGCTGTGACATCCATAC-3′ for osteocalcin, 5′-GCATTGACGCATCCAAACCC-3′ and 5′-CGTGGTAGGTCCAGCAAACAGTTAC-3′ for ADAMTS-5, 5′-ACTTTGTTGCCAATTCCAGG-3′ and 5′-TTTGAGAACACGGGGAAGAC-3′ for MMP-13, 5′-ACCCCAAGGACCTAAAGGAA-3′ and 5′-CCCCAGGATACCCTGTTTTT-3′ for Col 10, 5′-TTGCGACCATTGTTAGCCACATA-3′ and 5′-TCAGATCCATAGTGAAACCGCAAG-3′ for TRAP, 5′-CAGCAGAACGGAGGCATTGA-3′ and 5′-CTTTGCCGTGGCGTTATACATACA-3′ for Cathepsin K, 5′-AAAATGGCAGTGCGTTTAG-3′ and 5′-TTTGAAGGCAGTCTGTCGTA-3′ for β-catenin, 5′-TACAAGCTGGCTGGTGGGGA-3′ and 5′-GTCGCGGGTCTCAGGACCTT-3′ for NF-κB2, 5′-AGAACATCATCCCTGCATCC-3′ and 5′-AGTTGCTGTTGAAGTCGC-3′ for GAPDH, 5′-TGAGGAAGAAGCCCATTCAC-3′ and 5′ ACTTCTTCTCCCGGGTGTG-3′ for Osterix, 5′-GTCAACGGCACCAGCACCAA-3′ and 5′-GTAGCTGTATTCGTCCTCAT-3′ for BSP, 5′-GAAGTCAGCTGCATACAC-3′ and 5′-AGGAAGTCCAGGCTGTCC-3′ for Col 1. The presence of a single specific PCR product was verified by melting curve analysis and for each gene, the experiments were repeated three times (n = 3, respectively).

### Histology

Murine specimens from L2–L4 IVD and adjacent vertebral bodies were fixed in 4% paraformaldehyde, decalcified, dehydrated, cleared with dimethylbenzene, and were then embedded in olefin. At least 4 consecutive 6-μm sections were obtained from the sagittal planes, and then stained using hematoxylin and eosin (HE) for routine morphologic analysis. IVD structures were defined based on an instruction as previously reported^46^. Safranin O staining was used to evaluate proteoglycan change and TRAP staining for identifying osteoclasts. The morphology of the cartilage endplate, annulus fibrosus, and nucleus pulposus was examined using OsteoMeasure software (OsteoMetrics, Inc., Decatur, GA) and images were acquired with a light microscopic system (Olympus IX71, Olympus America Inc., Center Valley, PA).

### Immunohistochemistry

Seven IVD samples from patients with disc degeneration were harvested in this study, and Institutional Review Boards (IRB#2852 from Sutter Medical Center in California) approved the experiments. Informed consent was obtained from all subjects. Besides, IVD tissue from 4-, 6- and 9-month old WT mice were harvested and fixed in 4% PBS buffered paraformaldehyde at 4°C overnight. After the tissue was dehydrated and embedded in paraffin, 6-μm sections were cut. Thereafter, sections were deparaffinized by xylene immersion, rehydrated by graded ethanol, and treated with 0.1% trypsin for 30 minutes at 37°C. After blocking in 20% goat serum for 60 minutes at room temperature, sections from human IVD were incubated with anti-PGRN polyclonal antibody (1:100 dilution; Santa Cruz Biotechnology), and sections from indicated mice were incubated with anti-neoepitope of aggrecan (1:100 dilution; Millipore, Cat. No: AB8135), anti-phosphorylated IκB-α (pIκB-α) (1:100 dilution; Santa Cruz Biotechnology; Cat. No. SC-101713) or anti-β-catenin polyclonal antibody (1:100 dilution; Santa Cruz Biotechnology; Cat. No. SC-1496) at 4°C overnight, followed by incubation with a horseradish peroxidase–conjugated secondary antibody for 60 minutes at room temperature. The signal was detected using the Vector Elite ABC Kit (Vectastain; Vector).

### Western blot

Total IVD extracts of indicated ages from WT and PGRN−/− mice were homogenized and proteins were collected. 3 mice of each group were used in this experiment. For each mouse, protein extracts from different IVD segments were collected together for Western blot. Proteins were resolved on a 10% SDS-polyacrylamide gel and electroblotted onto a nitrocellulose membrane. After blocking in 5% nonfat dry milk in Tris buffer-saline-Tween 20 (10 mM Tris-HCl, pH 8.0; 150 mM NaCl; and 0.5% Tween 20), blots were incubated with polyclonal anti-PGRN, anti-phosphorylated IκB-α (pIκB-α), anti-iNOS or anti-β-catenin (diluted 1:1000) antibody for 1 h. After washing, the secondary antibody (horseradish peroxidase conjugated anti-rabbit immunoglobulin; 1:2000 dilution) was added, and bound antibody was visualized using an enhanced chemiluminescence system (Amersham Life Science, Arlington Heights, IL, USA).

### Histological scoring for degeneration of IVD

The degeneration of L3–L4 IVD was scored according to the classification system proposed by Boos et al^20^. This was a classification system for grading the histological features of age-related changes in the lumbar disc. Histological gradings were performed separately on nucleus pulposus (NP)/annulus fibrosus (AF), and endplate (EP). This classification system is based on an extensive semiquantitative histological analysis (NP/AF 0–22, EP 0–18, total 0–40). With this scoring system, a higher score indicates a more severe stage of disc degeneration. In the present study, all the sections underwent double blind examinations by 2 authors independently (Y. Z and B. R).

### Statistical analysis

The Statistical Package for Social Sciences version 17.0 (SPSS Inc, Chicago, IL) was used for standard statistical analysis including one-way ANOVA and Student's t-test. Statistical significance was achieved when a value of *P* < 0.05.
